# Editorial of Special Issue “The COVID-19 Pandemic and Bacterial Infections: Microbiological and Clinical Aspects”

**DOI:** 10.3390/microorganisms11041009

**Published:** 2023-04-12

**Authors:** Vincenzo Di Pilato, Daniele Roberto Giacobbe

**Affiliations:** 1 Department of Surgical Sciences and Integrated Diagnostics (DISC), University of Genoa, 16132 Genoa, Italy; 2 Department of Health Sciences (DISSAL), University of Genoa, 16132 Genoa, Italy; 3 Infectious Diseases Unit, IRCCS Ospedale Policlinico San Martino, 16132 Genoa, Italy

The emergence in late 2019 of the severe acute respiratory syndrome coronavirus 2 (SARS-CoV-2), the causative agent of the pandemic coronavirus disease 2019 (COVID-19), posed significant health challenges worldwide [1].

Overall, individuals infected by SARS-CoV-2 exhibited a wide range of clinical manifestations, primarily affecting the upper respiratory tract and necessitating admission to intensive care units (ICUs) in the most at-risk groups [2]. Hospitalized, severely ill COVID-19 patients can develop bacterial co-infections or super-infections, which could be associated with worse outcomes.

Most recent data from the literature suggest that although bacterial infections in COVID-19 occur with a low overall prevalence, some differences can be observed according to the patients’ population [3,4]; indeed, while the chance of bacterial co-infections is low in subjects with COVID-19 presenting in hospitals, a higher risk of secondary infections exists among patients admitted to ICUs [3].

The impact of bacterial infections on the severity of respiratory diseases, including the microbiological and clinical characteristics of such infections, remains understudied. As a consequence, knowledge gaps about the possible synergistic interactions between the SARS-CoV-2 infection and certain coinfecting bacteria or about the burden of antibiotic-resistant bacteria, considering the high antibiotics consumption observed among COVID-19 patients [3], exist.

This Special Issue on “The COVID-19 Pandemic and Bacterial Infections: Microbiological and Clinical Aspects” aims to broaden our understanding of the prevalence, incidence, and characteristics of bacterial infections in COVID-19 patients.

The research paper by Cohen et al. retrospectively assesses the rates and characteristics of pulmonary infections and the associated outcomes of ventilated COVID-19 patients (n = 93) using molecular syndromic assays [5]. The results show that most enrolled patients (68%) had ventilator-associated pneumonia (VAP), whose diagnosis was associated with poor patient outcomes, in contrast to community-acquired pneumonia (17%). Moreover, patients with VAP were older than those with community-acquired pneumonia (CAP) or those with no infection (68.5 vs. 57–59 years). Typical and expected organisms were associated with VAP (*P. aeruginosa* and *S. aureus*) and CAP (*H. influenzae*, *S. pneumoniae*, *M. catarrhalis,* and *E. cloacae*).

On the other hand, a rare case of pneumonia by *Hafnia alvei* was reported in a critically ill patient with a serious type 1 (hypoxemic) respiratory insufficiency, requiring extracorporeal membrane oxygenation [6]. In the former case report, Mendez et al. highlighted that owing to the immunocompromised status observed in COVID-19 disease, difficult-to-treat nosocomial respiratory co-infections by rare organisms could occur.

In this same line, Deghmane and Taha identified profound changes in the epidemiology of invasive bacterial infections following the emergence of the COVID-19 pandemic in France [7]. The results from this epidemiological investigation showed a drastic reduction in the circulation of *H. influenzae* and *N. meningitidis*, regardless of vaccine uptake, likely due to the implementation of strict social restrictions aimed at limiting the circulation of SARS-CoV-2.

Finally, in a retrospective observational cohort study, Ego et al. carried out a qualitative and quantitative analysis of sedative use in COVID-19 patients suffering from acute respiratory distress syndrome (ARDS) and in non-COVID patients who suffered from ARDS as a consequence of bacterial or viral infections and polytrauma [8]. Overall, COVID-19 patients with ARDS received a more frequent combination of multiple sedative drugs than non-COVID patients, resulting in a longer duration of mechanical ventilation and ICU stay.

In conclusion, the interplay between COVID-19 and bacterial co-infections and super-infections is complex in terms of both epidemiology and clinical impact. Certainly, despite the high rates of bacterial infections that have been reported in the most critically ill COVID-19 patients, the overall prevalence of bacterial infections in hospitalized COVID-19 patients remains low (4–6%) [9], strikingly contrasting with the high prevalence of antibiotic use reported by the same population (60–100% in most studies) [10,11]. This latter represents the clearest modifiable factor on which to intervene to reduce the unfavorable consequences of the COVID-19 pandemic on antibiotic resistance. Further efforts to mitigate this uncomfortable disproportion are warranted.
